# Health decline in prison and the effects of sporting activity: results of the Hessian prison sports study

**DOI:** 10.1186/s40352-023-00237-6

**Published:** 2023-08-29

**Authors:** Michael Mutz, Johannes Müller

**Affiliations:** Justus-Liebig-University Giessen, Kugelberg 62, 35394 Giessen, Germany

**Keywords:** Incarceration, Physical activity, Exercise, Health care, Life satisfaction

## Abstract

**Background:**

Previous studies about health in prisons conclude that incarceration has detrimental consequences for physical and mental health. It is associated with weight gain, decreased fitness, increased cardiovascular risks, and increased risks for mental illnesses, like depression and anxiety. This article examines the relationships between sports activity and health developments among prisoners. We analyze data from the Hessian Prison Sports Study, conducted in 12 prisons of the federal state of Hesse, Germany.

**Results:**

Based on quantitative survey data of 568 prisoners in regular custody, our empirical findings show that inmates perceive substantial health declines since incarceration. They report substantial decreases in general health (*d*=-0.52) and life satisfaction (*d*=-0.84) as well as an increased number of health problems (*d* = 0.71). However, sport has a potential to buffer this decline of health. Prisoners engaged in sports report a less negative development of their health compared to inactive prisoners. The greater the amount of time spent with sports activities, the better are the health trajectories found.

**Conclusion:**

Findings add to the public health and prison sport literature by demonstrating health benefits of sporting activities in a vulnerable population group that almost inevitably is exposed to manifold strains and burdens.

## Introduction

Studies from various countries point to a high number of incarcerated people with poor health or describe inmates’ health states as worse compared to the average level of non-incarcerated individuals (e.g., Binswanger et al., 2009; Hanssens et al., 2018; Heidari, Dickson & Newton, 2014; Trotter et al., 2018). Large-scale surveys from the U.S. indicate that between 39% and 43% of inmates in federal and state prisons suffer from a chronic medical condition (Wilper et al., 2009). They have an above average prevalence of diabetes mellitus, hypertension, asthma, arthritis, or HIV (Binswanger et al., 2009; Wilper et al., 2009). They also have higher risks of mental disorders (Yi, Turney & Wildeman, 2017), e.g. four times higher risks for depression or psychosis compared to the general population (Fazel & Baillargeon, 2011). Moreover, studies also point to a high prevalence of illegal drug misuse in prison (Carpentier et al., 2012; Keppler et al., 2010) and to suicidal tendencies (Byng et al., 2015).

A variety of reasons are discussed that may help to explain the poor health conditions among inmates. Firstly, imprisonment itself is a cause for chronic stress and suffering. Typical stressors include the loss of autonomy, personal belongings, heterosexual relationships, but also the separation from family and friends as well as being around fellow inmates who are prone to violence. Sykes (1958) has described these strains as the “pains of imprisonment” and his pioneering work has been widely acclaimed to this day, with extensions to his original concept being proposed time and again (Crewe, 2011; Hancock & Jewkes, 2011). Secondly, prisoners may not have access to adequate health care. For instance, a study reports that in U.S. federal and state prisons between 14% and 20% of prisoners with a persistent medical problem, do not receive timely medical examination (Wilper et al., 2009). Thirdly, prison management often marginalize health promotion (Woodall, 2016). Hence, the typical prison environment makes it difficult for prisoners to carry out health-promoting behaviors, like smoking cessation, healthy eating or physical activity in fresh air.

Given the fact that imprisonment in modernity aims at inmates’ reintegration into society, civilized forms of incarceration are supposed to avoid unnecessary suffering, which includes the duty to care for the health of the incarcerated. The World Health Organization (WHO) has identified prisons as a crucial setting for health-promoting interventions (WHO, 2021). The WHO’s prison health framework calls for better health surveillance, health promotion, and medical services. One particular aspect of health promotion is to allow prisoners to engage in physical activity and sports. There is ample evidence for physical activity’s role for health and well-being *outside of prisons* (e.g., Ekelund et al., 2019). Less clear, however, is the potential role of sporting activity for maintaining or improving health inside the prison.

Health benefits resulting from sporting activity should not be generalized all too quickly to the group of prisoners due to several peculiarities of the prison context. The sports on offer are limited with only few sports that can be practiced inside the prison walls. For instance, German prisons most frequently offer table tennis, soccer, volleyball and running groups as well as a small range of fitness activities (Müller & Mutz, 2023). Many prisoners lack the knowledge to exercise in a self-organized way and the cells and spaces provided offer few opportunities for informal sports. Moreover, sport in prison is much more strongly associated with perspectives of meaning that have little to do with health. Prisoners exercise to stage a stable body, feel strong and self-defensive or to express their masculinity (Martos-García, Devís‐Devís & Sparkes, 2009; Maycock, 2018; Müller & Mutz, 2022; Norman, 2017; Sabo, 2001). Finally, imprisoned men often start their sentences with unfavorable physical conditions for sports, e.g. because of addictions to substances (Stöver, 2017; Carpentier et al., 2012). Considering that health benefits of sport vary between different activities, are related to intensity, frequency and duration, are shaped by social contexts and depend on individual preconditions of those exercising (e.g., Oja et al., 2015; Vella et al., 2023; White et al., 2017), specific research on the relationship between sport and health in prisons is highly relevant.

The present study thus investigates the potential health benefits of sporting activity for prisoners. For analyzing this relationship, we use data from the *Hessian Prison Sport Study* that represents the population of males imprisoned in the German federal state of Hesse. This data set includes prisoners’ answers regarding their current health status as well as their responses to retrospective questions, referring to the time before imprisonment. We use these data to explore two research questions: Firstly, we describe inasmuch key indicators of health systematically change since the beginning of imprisonment. Secondly, we analyze the role of sporting activity in prison, i.e. if sport helps to maintain or even improve health during incarceration. Before that, however, the article proceeds with a review of literature on health trajectories in prison as well as the benefits of prison sport for physical and mental health.

## Health trajectories of prisoners

Several studies examined health trajectories of prisoners. Some of them focus on the effect of incarceration on weight status. For instance, quantitative studies from France and Italy conclude that incarceration is associated with a significant increase of Body Mass Index (BMI) and a higher rate of obesity (Lagarrigue et al., 2017; Rocca, 2018). Choudhry et al. (2019) report an increase of BMI in a quantitative study of 251 prisoners in the UK. This increase occurs mainly six to 12 months after incarceration. A recent review (Bondolfi et al., 2020) identifies a mean increase of 5.3 kg and change of BMI of 1.8 kg/m^2^ in the first two years of imprisonment. Two years after entering the prison, this increase reaches a ceiling. Findings of the review also suggest trends towards increased blood pressure and hypertension.

Other studies focus on female prisoners. A Swiss study with 60 incarcerated adult women points out that 68% report that their self-assessed health had worsened since incarceration (Augsburger et al., 2022). Qualitative studies also show that imprisoned women perceive that their mental health declines in prison (Douglas, Plugge & Fitzpatrick, 2009). They attribute this decline to the separation from families, living in close proximity with other suffering individuals, unhygienic facilities, disempowerment and a health negating lifestyle and diet. Studies with a particular focus on the prison environment conclude that prisons reduce health by fostering disempowerment and deprivation (De Viggiani, 2007). Particularly long periods of isolation with little mental stimuli contribute to poor mental health and intense feelings of anger, frustration and anxiety (Nurse, Woodcock & Ormsby, 2003). However, Alves et al. (2016) put a different result forward when they conclude that some female inmates, for instance those with either a chronic disease or a drug addiction, receive better medical treatment and therapies in prison than outside of prisons. They conjecture that health conditions may improve when individuals lived under adverse conditions or extreme poverty before their incarceration.

Some papers foreground the duration of sentence and its impact on health. Constantino et al. (2016) shows that male prisoners, who are incarcerated between one and nine years, are 0.55 times less likely to experience stress symptoms compared to those in their first year of imprisonment. A review also concludes that reception into prison results in increased levels of psychiatric symptoms, which then soften over time (Walker et al., 2014). These findings are in line with findings from Crewe et al. (2020), who highlight the “entry shock” in the first weeks and months of a longer prison sentence. Characteristics of this initial period are acute stress reactions and strong emotions of despair that exceed the ‘normal’ stress of imprisonment. They conclude that there is “little ostensible indication that the problems of such sentences accumulate or worsen according to time served”, but in fact, strains are greatest at the beginning of the prison sentence and then decrease later on (Crewe et al., 2020, p.84).

Taken together, these findings suggest that health outcomes are more likely to be unfavorable following incarceration. However, it is not yet possible to say with certainty whether there is a gradual deterioration of health in the course of imprisonment or a rapid deterioration at the beginning of the sentence. The results on physical health point more in the first direction, i.e., a gradual deterioration. The results on mental health tend to indicate that the greatest problems prevail at the beginning of imprisonment. However, the following research findings suggest that physical activity and sport in prison could work as a possible remedy.

## Health benefits of sporting activities in prison

Sociological accounts on prison life note that a large number of inmates engage in organized sports and exercise or in informal strengths training, while being incarcerated (Harvey, 2012; Maycock, 2018; Norman, 2017; Norman et al., 2021). Some qualitative studies highlight that sporting activity is beneficial for psychosocial health, as it seems to buffer stress, frustration, and loneliness and is a temporary escape from monotony (Martos-García, Devís‐Devís & Sparkes, 2009; Meek and Lewis, 2014; Müller & Mutz, 2019). Others describe sport as a means to release aggression and to find a short-term relief from fears (Crewe et al., 2014). This escape and relief function of sport is likely to be important for mental health, especially against the background of the strains described above.

These findings from qualitative studies, which reflect prisoners’ own perspectives, complement very well with results from intervention studies. After taking part in a nine months long, supervised exercise program, male Italian prisoners showed a better mental well-being (Battaglia et al., 2015). Another intervention study with prisoners with anxiety symptoms found a significantly greater reduction in anxiety after an interval exercise training compared to non-participants (Legrand, Ory & Herring, 2020). Other interventions carried out in British and Swedish prisons made use of yoga (Bilderbeck et al., 2013, 2015; Kerekes et al., 2017): They also document mental health benefits, for instance, increased positive affect and reduced stress levels. A review of 14 intervention studies that used a variety of different sport and exercise activities concludes that these programs help to improve prisoners’ well-being and reduce stress (Woods, Breslin & Hassan, 2017).

In addition to the function of sport and exercise for psychological health of inmates, a variety of studies and reviews also address physical health benefits of prison sports. Several intervention studies examined the effects of three- to four-month long supervised exercise programs on the physical fitness and health of detainees (Bueno-Antequara, Oviedo-Caro & Munguía-Izquierdo, 2019; Cashin et al., 2008; Pérez-Moreno et al., 2007). These studies show that even such a relatively short training intervention can help to improve incarcerated males’ physical fitness in general and cardiorespiratory fitness in particular. Recent reviews of interventions and randomized controlled trials buttress these results, concluding that supervised physical activity and exercise programs in prison improved health parameters and reduced risk factors for cardiovascular diseases such as participants’ blood pressure and cholesterol levels (Mohan et al., 2018; Papa et al., 2021; Sanchez-Lastra et al., 2019). Additional findings indicate that exercising in prison can reduce weight gain (Johnson et al., 2019): Inmates who engaged in at least 60 min of daily physical activity gained less weight (4.5 kg) since incarceration than inactive inmates (8.3 kg).

Most of the previous research used intervention designs with relatively small samples of usually 20 to 60 individuals and offered exercise programs that often lasted only a few weeks. Considering this, the current study complements the existing body of research, because it uses a larger and representative sample of prisoners to analyze the relationship between sports activity and health. Moreover, we do not solely look at formal, instructed sports activities, but also at informal activities, because the latter ones also yield health benefits. Precisely, we assume that changes in physical and mental health in prison are unfavorable overall, but supposedly less negative when prisoners spent sufficient amount of time with sports activity.

## Methods

### Sample and data collection

The *Hessian Prison Sport Study* (HePSS; Müller & Mutz, 2023) uses quantitative survey data collected in prisons located in the federal state of Hesse, Germany. Hesse is a typical, medium-sized German state with almost 6 million inhabitants, an urban metropolitan region around Frankfurt am Main, but also structurally weak, rural regions. *HePSS* aimed to explore the relationship between sporting activity and some key aspects of health among imprisoned individuals. Data were collected in all 12 Hessian prisons that incarcerate male adult prisoners. As the study intent was to address male prisoners in regular custody, detention centers for juveniles, women prisons, prison units for pre-trial detainees as well as special units for preventive detention of prisoners particularly at risk of recidivism after actual imprisonment were excluded. Overall, the state of Hesse has 2,467 incarcerated male adults in regular custody.^1^

The Hessian prisons differ in their size and include larger prisons (with approx. 450 prisoners) and smaller prisons (with < 30 prisoners). In smaller prisons, we decided to invite all prisoners to participate in the survey whereas in larger prisons, we applied a cluster sampling approach (e.g., Thompson, 2012) and randomly selected single detention units within the prison. This procedure is reasonable, given that detention units have a heterogeneous composition in all Hessian prisons. Moreover, after consulting the prison management and staff, we excluded all prisoners from the survey whose German language proficiency was too low for reading and understanding the questionnaire and the consent form. Hence, all randomly selected prisoners with sufficient German language skills (N = 1,672) were invited to take part in the survey.

These prisoners received the consent form and a questionnaire. Prison staff informed them that participation was voluntary and non-participation not associated with any disadvantage. Prisoners who decided to participate then filled out the questionnaire in their cells. Afterwards, they placed the completed questionnaire in a closed envelope and into a sealed ballot box. This ensured that all information was anonymous and that prison staff could not view the answers. The study and all procedures described received approval from the local ethics committee of the Faculty of Psychology and Sport Science of the University of Giessen (Reg. No. 2021-0035).

Overall, we received 568 completed questionnaires (response rate: 34.0%). To account for minor variations between smaller and larger prisons regarding response rates, we calculated probability weights. These weighting factors adjust the composition of the sample for the relations in size that exist across prisons. In other words, the weighted *s*ample represents large and small prisons exactly according to their share of the total Hessian prison population.

Our final sample includes adult prisoners of all age groups (*M* = 40.8; *SD* = 10.9; *min* = 22; *max* = 83) and with various educational backgrounds (no formal education = 12.1%; lower secondary degree = 38.0%; medium secondary degree = 31.3%; higher secondary degree = 11.3%; academic degree = 7.3%). Moreover, the sample consists of prisoners who have been in prison for relatively short periods of less than 12 months (28.7%) as well as prisoners who have already served longer sentences of up to two years (30.6%), up to five years (21.7%) or longer than five years (19.0%). In the sample, 44.3% are multiple offenders who are serving at least their second prison sentence, while 55.7% are first-time prisoners.

### Measures

*Subjective Health.* HePSS assessed health with three indicators: self-rated general health, life satisfaction, frequently occurring health issues. These measures capture key aspects of physical and psychological health. (1) Self-rated general health (“*Please rate your overall state of health: Is your health…*”) is a key indicator of a person’s overall health condition, recommended for surveys because of its briefness, simplicity and validity (Lazarevič, 2019). Respondents answered this question on a 10-point Likert scale from 1= “*very poor*” to 10= “*excellent*”. (2) Satisfaction with life (“*How satisfied are you overall with your life in general*”) is a valid single-item measure of subjective well-being (Cheung & Lucas, 2014). Again, respondents answered this question on a 10-point Likert scale with answer categories from 1= “*completely unsatisfied*” to 10= “*completely satisfied*”. (3) The measure for physical health issues consisted of a list of symptoms for each of which respondents indicated how often they experienced it. The list of symptoms included (1) headache, (2) back pain, (3) stomach pain, (4) knee or joint pain, (5) toothache, (6) sleep problems, (7) lack of drive. For each health problem, answers ranged from 1= “*never*” to 6= “*several times a week*”. We calculated a sum score of those symptoms that respondents reported experiencing at least “*several times a month*”.

The corresponding questions were asked twice: firstly, with a reference to the time before incarceration (“*Please think back to the time before imprisonment…*”) and secondly, with reference to the present time, i.e. during incarceration. Scholars consider retrospective pretest-posttest designs (RPP) as a serious alternative to traditional pretest-posttest designs, particularly because changes in the frame of reference among the respondents are avoided (Little et al., 2020). Hence, we use retrospective health data to assess the initial health status from the time before incarceration and compare these with data from the time of imprisonment.

The health indicators used in this study correlate with each other, which is expected. The correlations have a moderate size: General health during imprisonment and life satisfaction correlate with 0.54 (Pearson’s *r*, *p* < .01) and general health and health complaints with 0.55 (*p* < .01). Life satisfaction and health complaints correlate with − 0.34 (*p* < .01).

*Sport and exercise activity*. We assessed involvement in sport and exercise activity with a question that inquired about the amount of time per week invested in respective activities (“*Please think about the last few weeks: How much sport and exercising did you do per week?*”). The questionnaire mentioned playing soccer, taking part in a running group or fitness training as examples to make sure that the respondents understood the question similarly. Four groups are compared in the analyses: Prisoners who reported to “*never*” exercise or play sport (26.0%), those who engage in sporting activity up to 1.5 h per week (21.1%), between 2 and 4 h per week (27.8%) and 5 or more hours per week (25.0%).

*Controls*. We control for age (in years) and the person’s highest educational degree in all analyses. Moreover, we also control for screen time. Scholars often use screen time as a proxy for sedentary behavior (O’Donoghue et al., 2016), which is an independent risk factor for health (Tremblay et al., 2010). However, in the context of the prisons studied here, screen time is exclusively television time, because often no other screen-based devices and media are available or allowed. The analyses distinguishes four groups: Prisoners with a daily screen time of less than 2 h (13.8%), approximately 2 h (18.3%), between 3 and 4 h (46.6%) and >4 h per day (21.3%).

### Analytical approach

We calculate linear regression models with each one of the health indicators as the dependent variable (with reference to the period of imprisonment) and the same health indicator (with reference to the period before imprisonment) as a covariate. Hence, the models assess health during imprisonment adjusted for the health status before imprisonment. Following the key arguments elaborated by Little et al. (2020), we see the strength of this design in the consistent frame of reference each respondent uses for their replies and the lack of a response shift bias, respectively. Sporting activity during incarceration is included as the key predictor into the models, which additionally also control for important confounders, i.e. age, education as well as screen time in prison as a proxy for sedentariness. All analyses use weighting factors (as described above) and are conducted with IBM SPSS 28.

## Findings

### Self-rated overall health

**Fig. 1:**
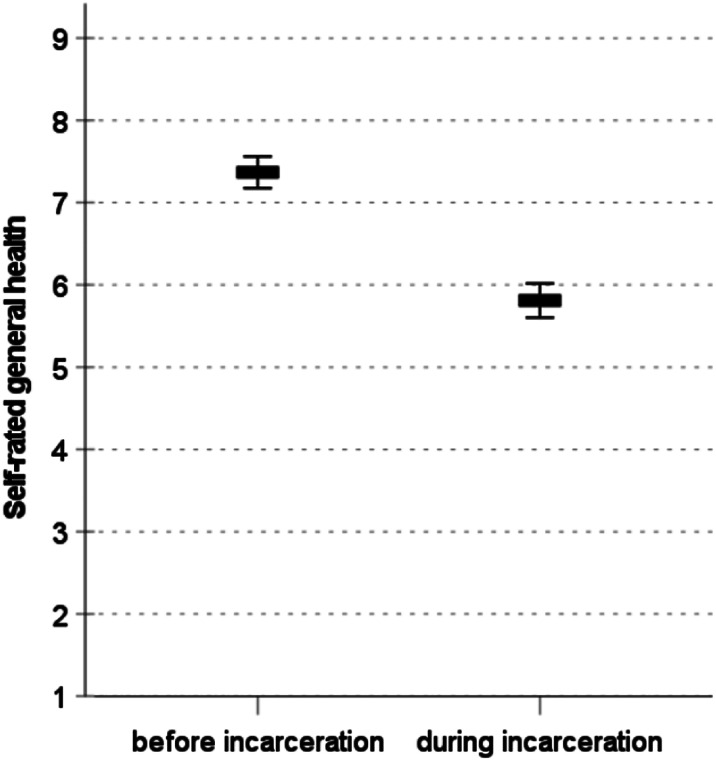
Self-rated general health among inmates before and during incarceration. Graph shows means and 95% confidence intervals. The difference of the means is significant with *p* < 0.001

Inmates report a substantial decline in their overall health after incarceration (Fig. 1). Compared to the time before imprisonment, they rate their health as 1.56 points less favorable on a 10-point rating scale (*M*_t1_=7.37; *M*_t2_=5.81; *Diff*=-1.56; *d*=-0.52; *p* < .01). In a regression model that accounts for the differences in self-rated overall health before incarceration, sporting activity is a significant predictor for overall health during incarceration (Table 1, column 1). Prisoners who engage in 2 to 4 h sporting activity per week rate their overall health during incarceration 0.99 units better (on a 10-point scale) compared to prisoners who do not engage in sports. Prisoners who engage in > 4 h sporting activity per week rate their overall health even better, with a 2.12 unit difference compared to prisoners who do not engage in sports. Both effects are significant with *p* < .001. Screen time, length of sentence, age and education are not significantly associated with self-ratings of general health in prison.

**Table 1:** Regression models for three health indicators during incarceration

	General health		Life satisfaction		Health issues	
	*b*	*p*	*b*	*p*	*b*	*p*
**Recall questions (before incarceration)**						
General health	**0.17**	**< 0.001**	--	--	--	--
Life satisfaction	--	--	**0.10**	**0.018**	--	--
Health issues	--	--	--	--	**0.41**	**< 0.001**
**Sporting activity in prison** (Ref. no sports)						
up to 1.5 h/week	0.34	0.233	0.25	0.442	-0.20	0.414
between 2 and 4 h/week	**0.99**	**< 0.001**	**0.88**	**0.005**	**-0.54**	**0.026**
5 h/week or more	**2.12**	**< 0.001**	**1.31**	**< 0.001**	**-0.80**	**< 0.001**
**Screen time in prison** (Ref. <2 h/day)						
appr. 2 h/day	0.26	0.464	0.42	0.300	-0.49	0.109
between 3 and 4 h/day	-0.16	0.615	-0.32	0.362	0.46	0.085
5 h/day or more	-0.67	0.059	0.10	0.800	0.23	0.455
**Length of sentence to date** (Ref. <1 year)						
between 1 and 2 years	-0.16	0.538	-0.58	0.053	0.41	0.074
between 2 and 5 years	-0.28	0.340	-0.03	0.933	0.30	0.245
5 years and longer	-0.06	0.860	-0.27	0.464	0.14	0.612
**Socio-demographics**						
Age	0.01	0.320	0.01	0.293	-0.02	0.085
Education	-0.15	0.115	**-0.24**	**0.029**	0.08	0.339
**Model fit (R²)**	0.175		0.079		0.129	

### Life satisfaction

**Fig. 2:**
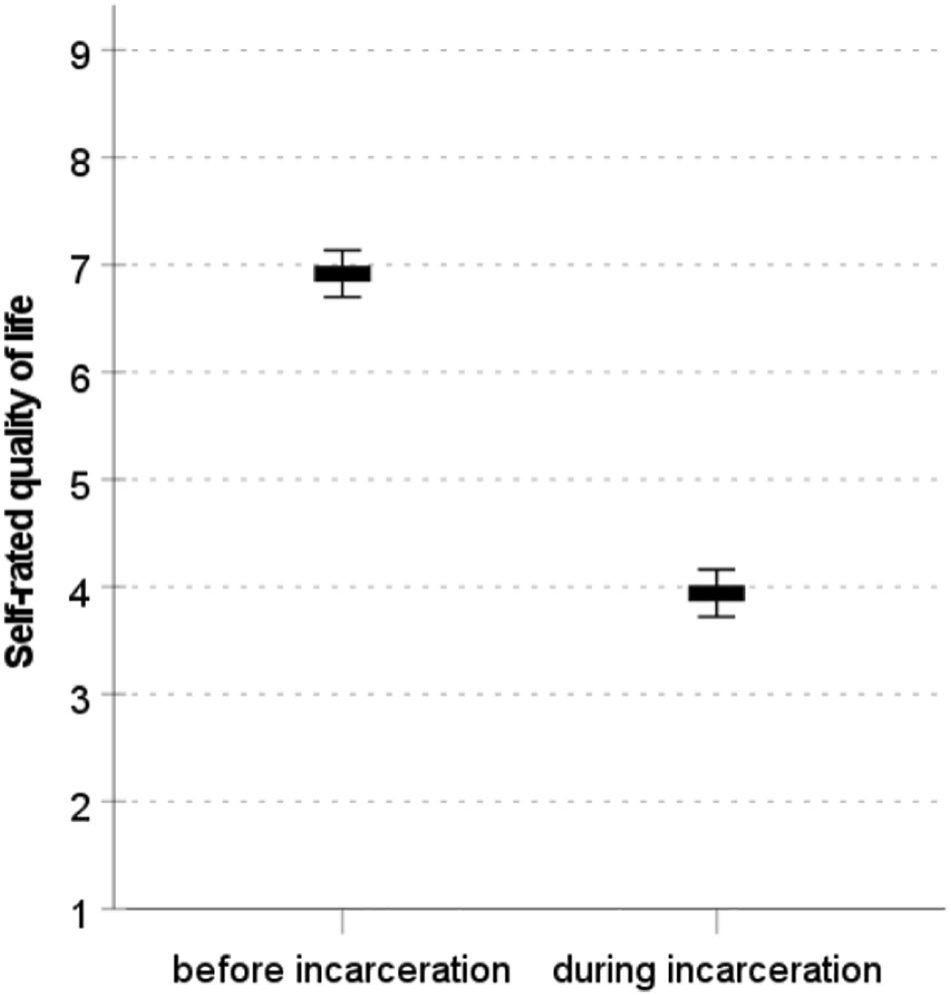
Life satisfaction ratings among inmates before and during incarceration. Graph shows means and 95% confidence intervals. The difference of the means is significant with *p* < 0.001

Inmates regard their life as less satisfying in prison compared to the time before incarceration (Fig. 2). The decline by almost 3 points on a 10-point rating scale is of substantial magnitude (*M*_t1_=6.92; *M*_t2_=3.94; *Diff*=-2.98; *d*=-0.84; *p* < .01). Moreover, the average life satisfaction scores in the Hessian prisons is very low, comparable to mean levels known from the poorest regions and most fragile states in the world (Veenhoven, 2022). In a regression model that accounts for the differences in life satisfaction before incarceration, sporting activity is a significant predictor for satisfaction with life during incarceration (Table 1, column 2). Prisoners who engage in 2 to 4 h sporting activity per week rate their life 0.88 units better (on a 10-point scale) compared to prisoners who do not engage in sports (*p* < .01). Prisoners who engage in > 4 h sporting activity per week rate their life 1.31 units better compared to prisoners who do not engage in sports (*p* < .001). Screen time, length of sentence, and age are not significantly associated with life satisfaction during imprisonment, while education has a negative effect.

### Health issues

**Fig. 3:**
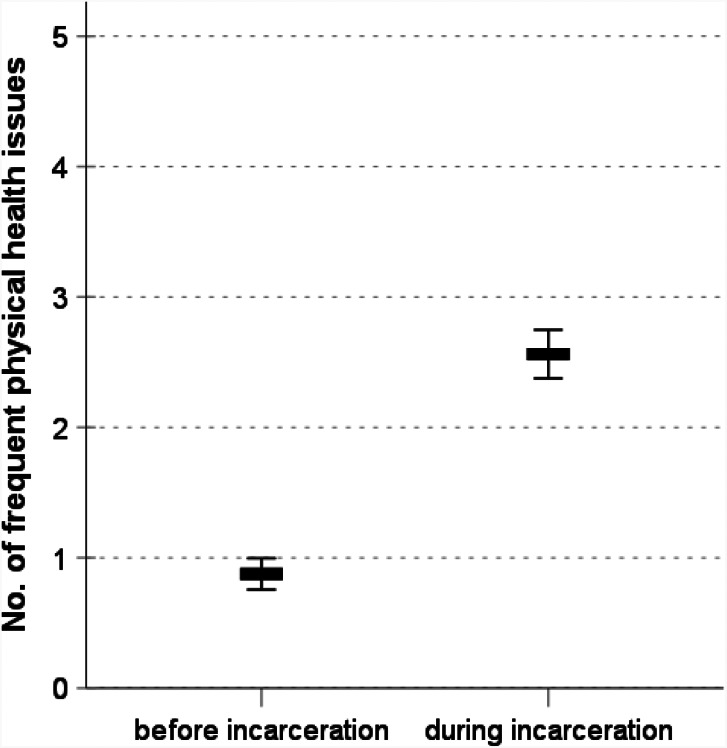
Physical health issues among inmates before and during incarceration. Graph shows means and 95% confidence intervals. The difference of the means is significant with *p* < 0.001

Prisoners report a greater number of frequently occurring health problems, like headaches or back pain, after their incarceration (Fig. 3). Compared to the time before imprisonment, they report an increase of 1.61 health complaints, on average, that they frequently experience (*M*_t1_=0.78; *M*_t2_=2.39; *Diff* = 1.61; *d* = 0.79; *p* < .01). Again, sporting activity is a significant predictor in a regression model that also accounts for the differences in health complaints before incarceration (Table 1, column 3). Prisoners who engage in 2 to 4 h sporting activity per week report 0.54 fewer health issues compared to prisoners who do not engage in sports (*p* < .05). Prisoners who engage in > 4 h sporting activity per week report 0.80 fewer health issues compared to prisoners who do not engage in sports (*p* < .001). Screen time, sentence length, age and education are not significant predictors of health issues during imprisonment.

## Discussion and conclusion

As a large scale prison sport study, *HePSS* aimed at exploring the relationship between sporting activity and health in German prisons. The study should shed light on the extent to which key indicators of health systematically change since the beginning of imprisonment and the potential role of sporting activities for maintaining health inside the prison. In this regard, our data first shows that inmate perceptions of their health decrease since incarceration. Precisely, inmates report substantial declines of their general health and life satisfaction as well as an increased number of health problems. The effect sizes for life satisfaction (*d*=-0.84) and health problems (*d* = 0.71) are large; for self-rated general health the effect is of moderate size (*d*=-0.52) (Cohen, 1992). These findings indicate that imprisonment does not pass by without strongly affecting imprisoned men psychologically and physically. Thus, incarceration proves to be a threat to inmates’ health. These findings fit in well with previous studies that also reported health declines in the course of incarceration (e.g., Augsburger et al., 2022; Bondolfi et al., 2020; Douglas et al., 2009; Nurse et al., 2003; Walker et al., 2014).

Secondly, our study suggests that sport has a potential to buffer or counteract the substantial decline of health during incarceration. Thus, sport in prison appears not only as a strategy for coping with incarceration (e.g., Müller & Mutz, 2019), but also a valuable resource for health promotion. In his classic work on total institutions, Goffman (1961) already pointed out that sport and exercise activities can help individuals in these institutions to endure psychological stress associated with the constant supervision and personal degradation. Although Goffman does not explicitly address health, his remarks certainly are relevant for mental health at least. Consistent with our findings, other studies have also pointed to benefits of prison sports for psychological and physical health (Woods, Breslin & Hassan, 2017; Battaglia et al., 2015; Sanchez-Lastra et al., 2019). However, our study puts these effects in relation to overall developments of health during incarceration. In this context, it becomes clear that prison sport is indeed beneficial to health, but this benefit does not lead to an improvement of health conditions, but rather to a less negative development. Additionally, our findings show that a greater amount of time spent with sports activities relates to better overall health, higher life satisfaction and fewer health issues. Hence, the amount of time spent in sporting activity is an important factor in counteracting health decline in prison.

Except for the strong health effects of prison sports, we find no effects for screen time and the length of imprisonment. In our study, long daily television time of three hours or more are quite common. These sedentary periods, however, do not consistently correlate with any of the health outcomes studied here. This may be the case because inmates are required to work and much of this work is of manual nature. Hence, most of the prisoners achieve a minimum of light physical activity on workdays, so that even if they watch a lot of television, they are somewhat active over the course of the day. However, the association between sedentariness and health was not the main concern of this study, so future work could certainly survey prisoners’ inactive periods in more detail and go beyond the measure of television viewing used here. In addition, the length of the imprisonment was largely uncorrelated with health. This is somewhat surprising, because previous work pointed to either a gradual decrease in health (Augsburger et al., 2022) or a sharp decline in the first year of captivity (Constantino et al., 2016). The present research does not support these suggestions, but instead tend to indicate that no systematic relations exist between health and the length of imprisonment.

With these key findings, this research also opens up fruitful avenues for further studies on health and sport in prison. Studies could focus, for instance, on the interdependence of worsened physical and psychological health or on the further development of health after release. Particularly the latter perspective would allow insights into the long-term health consequences of imprisonment. The strong decline of health found in this study could either be temporary as long as an individual is incarcerated or could persist permanently, also affecting life after incarceration (as suggested by Wildeman and Wang, 2017). With regard to the health benefits of prison sport, future studies could compare individual types of sport in a more differentiated way. In addition to the amount of time spent on sporting activities that we focused on, different forms and types of sport could yield differential effects.

As a practical consequence, findings of this study highlight the importance of implementing regular sports activities in prison. In Germany, prison laws dictate that inmates should have the opportunity to engage in sports, which is an important requirement in view of our findings. However, only a fraction of prisoners takes part in organized prison sports. We therefore see it as a necessity that sport offers reach more prisoners. For that to be possible there would have to be more and also more diverse sports offerings, as well as better sports infrastructure. In particular, there seem to be too few sports programs for older, inexperienced, unfit or chronically ill prisoners. Decision-makers in prisons could thus take findings provided in this study and in previous studies to advocate for an improvement of sports infrastructure within prisons. Only if prisons are equipped with appropriate indoor and outdoor sports facilities and provide prisoners sufficient opportunities to use these facilities, they can fully exploit the health benefits of sport. This is especially important in view of the current WHO public health strategy that explicitly address prisons (WHO, 2021). Besides the health benefits focused in this study, sport programs may also have positive side effects for the social climate in prisons and may facilitate the reintegration of prisoners into society (Meek & Lewis, 2014).

As a matter of course, our study is not free from limitations: Firstly, our study group exclusively includes prisoners with sufficient German language skills. It is unclear whether the health developments among non-German-speaking prisoners differ, especially in light of the fact that a large proportion of them presumably did not live permanently in Germany before their imprisonment. Secondly, our findings refer exclusively to male prisoners in closed prisons. We cannot assess the extent to which health changes among women, adolescents or inmates with less serious offences and less lengthy sentences, who are allowed to spend them in the open prison setting. Thirdly, the federal state of Hesse operates prisons of different size with the smallest prisons having a capacity of less than 30 individuals, while the largest prison holds close to 400 people. It can be conjectured though that prison size could influence the health status of prisoners, whereby health could deteriorate to a greater extent in larger prisons. Some previous studies reported an increase of social problems, such as physical victimization, in larger prisons (Caravaca-Sánchez et al., 2019; Wooldredge & Steiner, 2013) and this may also negatively affect individual health trajectories. Finally, the retrospective pretest-posttest design could attract criticism. Although there are strong arguments that retrospective data are valid (Little et al., 2020), these data and respective research designs are nevertheless not as established as pretest-posttest designs. One strength of these retrospective designs is that they avoid a “response shift bias”, i.e. that the internal reference frame for making personal judgements changes between measurement points (Pratt, McGuigan & Katzev, 2000). A weakness is a potential recall bias, occurring when initial conditions are judged in a distorted way because they cannot be remembered accurately. This bias is likely to be all the greater the longer the point in time has passed about which a retrospective statement is to be made. However, asking offenders about their physical and psychological health *before* their imprisonment is hardly feasible, so that few alternatives to our study design exist.

## Data Availability

The data that support the findings of this study are available from the corresponding author upon reasonable request.

## Footnotes

1 Personal communication with the Hessian Criminological Service, data are from February 2022.
