# Correction: Health decline in prison and the effects of sporting activity: results of the hessian prison sports study

**DOI:** 10.1186/s40352-023-00251-8

**Published:** 2023-12-04

**Authors:** Michael Mutz, Johannes Müller

**Affiliations:** Justus-Liebig-University Giessen, Kugelberg 62, 35394 Giessen, Germany

**Correction: Health Justice 11, 34 (2023)**

10.1186/s40352-023-00237-6

This note is to inform readers that an incorrect earlier version of the article was erroneously published due to an error by the publisher. In the original article (Mutz & Müller, 2023), Figs. 1, 2 and 3 are not displayed graphically correct (corrupted) in the html version online. Though figures in the pdf version are displayed correctly.

The html version of the figures in the original article (Mutz & Müller, 2023) has been corrected.
